# Fucoxanthinol Mitigates the Cytotoxic Effect of Chlorpyrifos and MPTP on the Dopaminergic Differentiation of SH-SY5Y Human Neuroblastoma Cells

**DOI:** 10.1007/s12031-025-02342-7

**Published:** 2025-04-08

**Authors:** Ekramy M. Elmorsy, Ayat B. Al-Ghafari, Huda A. Al Doghaither, Mona M. Elghareeb, Mouhamed Alsaqati

**Affiliations:** 1 Center of Health Research, Northern Border University, 91431 Arar, Saudi Arabia; 2 Department of Biochemistry, Faculty of Science, King Abdulaziz University, 21589 Jeddah, Saudi Arabia; 3 Experimental Biochemistry Unit, King Fahd Medical Research Center, King Abdulaziz University, 21589 Jeddah, Saudi Arabia; 4 Department of Physiology, Faculty of Veterinary Medicine, Mansoura University, Mansoura, 35516 Egypt; 5 School of Pharmacy, Faculty of Medical Sciences, Newcastle University, King George VI Building, Newcastle-Upon-Tyne, NE1 7RU UK; 6 Translational and Clinical Research Institute, Newcastle University, Newcastle-Upon-Tyne, UK

**Keywords:** Fucoxanthinol, Cytotoxic effect, MPTP, Chlorpyrifos, Parkinson’s disease

## Abstract

This study investigates the neuroprotective effects of fucoxanthinol (FXL) against the toxic activities of two compounds known to induce neurotoxic effects in humans and animals. MPTP (1-methyl- 4-phenyl- 1,2,3,6-tetrahydropyridine) induces Parkinson’s disease (PD)-like phenotypes by inhibiting mitochondrial complex I in dopaminergic neurons. Chlorpyrifos (CPF), another neurotoxic agent, is associated with acute and long-term neurotoxicity primarily through acetylcholinesterase (AChE) inhibition. FXL demonstrated the ability to reverse the neurotoxic effects of CPF and MPTP in SH-SY5Y dopaminergic neuronal cell models. Treatment with FXL enhances mitochondrial function in SH-SY5Y cells exposed to CPF and MPTP, as demonstrated by increased levels of Adenosine triphosphate (ATP), mitochondrial membrane potential (MMP), mitochondrial complexes activities, and oxygen consumption rates, pyruvate dehydrogenase (PDH) activities, and mitophagy pathways. This improvement highlights FXL’s ability to counteract the mitochondrial dysfunction induced by these neurotoxic agents. Additionally, FXL reduces oxidative damage and enhances cell viability. At the molecular level, the neuroprotective effects were also associated with the modulation of apoptotic cell markers, including Bcl- 2 and the oxidative damage markers. Molecular docking data further support the outcomes of our in vitro studies. Multivariable analysis highlights the neuroprotective effects of FXL. These findings indicate the potential of FXL to mitigate CPF- and MPTP-induced neurotoxicity, suggesting its promise as a therapeutic agent for managing neuronal damage observe in PD.

**Supplementary Information:**

The online version contains supplementary material available at 10.1007/s12031-025-02342-7.

## Introduction

Presently marketed insecticides provide excellent efficacy against pests, minimal environmental accumulation, and minimal harm to humans. Organophosphorus insecticides (OP) are the most widely used types of insecticides. One of the most used OP in the world is chlorpyrifos (O–O-diethyl-O- {3, 5, 6 trichloro- 2-pyridyl}- phosphorothioate; CPF) (Foong et al. 2020; Kaushal et al. 2021). Its primary hazardous metabolite; CPF-oxon (CPFO) is produced by the biotransformation of CPF by cytochrome P450 (CYP) enzymes, and CPFO is inactivated by A-esterase paraoxonase1 (PON- 1) and B-esterase carboxylesterases (CES) (Moser and Padilla 2016; Nandi et al. 2022).

By blocking acetylcholinesterase (AChE) function, chlorpyrifos (CPF) is known to cause acute and long-term neurotoxicity in humans and animals (Abou-Donia and Lapadula 1990). In addition to inhibiting AChE and generating in vivo toxicity, CPF may cause other actions in exposed cells. As a result, current studies have concentrated on determining the toxicity of CPF without considering how it affects AChE inhibition. According to both in vitro and in vivo research, CPF, even in low concentrations, caused oxidative stress (Ki et al. 2013), mitochondrial dysfunction (Elmorsy et al. 2022), disrupted neurotransmission (Slotkin and Seidler 2007), prevented nervous system cells from replicating (Qiao et al. 2001), disrupted neuronal differentiation (Yang et al. 2008), and caused neurobehavioral changes such as a decrease in children’s Mental Development Index scores and a weakness in psychomotor function. CPF and its metabolite, CPFO, can also cause apoptosis in various cell types, including neurons (Park et al. 2013; Caughlan et al. 2004).

When given to animals, MPTP (1-methyl- 4-phenyl- 1,2,3,6-tetrahydropyridine) was discovered to cause circumstances like Parkinson’s disease (PD). MPTP is a very lipophilic substance that quickly crosses the blood–brain barrier and is converted into the powerful neurotoxin MPP + in astrocytes by monoamine oxidase-B (Dauer and Przedborski 2003). The organic cation transporter- 3 allows astrocytes to discharge MPP + into the extracellular area (Cui et al. 2009). A dopamine transporter may then carry it into nearby dopaminergic neurons, concentrating in microglia and inhibiting the mitochondrial complex I of the mitochondrial electron transport chain, leading to oxidative stress and ATP depletion (Zeng et al. 2018). However, according to a study, MPP + -induced dopaminergic neuron death does not require mitochondrial complex I inhibition (Choi et al. 2008).

Fucoxanthin (FX) is the most prevalent marine brown algae carotenoid. It has many beneficial qualities against diabetes, obesity, cardiovascular disease, cancer, and neurological illnesses because of its distinct chemical structure (Bae et al. 2020). FX has been demonstrated in recent research to be effective in preventing several mechanisms underlying neurological diseases such as amyloid protein aggregation, inflammation, reactive species oxidative damage, neuronal apoptosis, as well as altered neurotransmission in various models for neurological disorders such as mental illnesses, Alzheimer’s disease (AD) (Xiang et al. 2017), PD (Sun et al. 2020; Paudel et al. 2019), and acute brain injury (Jiang et al. 2019). FX is hydrolyzed via the digestive enzymes to fucoxanthinol (FXL), which is then further transformed into amarouciaxanthin A in the liver and alimentary tract (Asai et al. 2004). After human volunteers were orally administered kombu algae containing FX, its bioavailability was examined. The results revealed the presence of FXL in human plasma, while FX itself was not detected (Hashimoto et al. 2012).

Parkinson’s disease (PD) is a neurodegenerative disorder characterized by dopamine dysfunction and impaired motor control. While the exact mechanisms underlying the disease’s pathology are not fully understood, it is widely believed that mitochondrial disruption, oxidative damage, and apoptosis play significant roles in its development mechanisms in the development of sporadic PD (Compagnoni et al. 2020). Therefore, the current study investigates the neurotoxicity of CPF and MPTP and the potential neuroprotective role of FXL using the differentiated dopaminergic (DA) neuron cell model and considering their relative impacts on cellular bioenergetics and oxidative damage.

## Materials and Methods

### Preparation of CPF, MPTP, and FXL Stock Solutions

All chemicals used in the current study were obtained from Sigma-Aldrich (Poole, UK). For further experiments, CPF and MPTP were dissolved in DMSO as a stock solution (100 mM). FXL was dissolved in DMSO (20 mM) before being added to the media, with the final DMSO concentration in all assays kept lower than 0.1%.

### The SH-SY5Y Cell Differentiation Culture Conditions

The SH-SY5Y cells were acquired from the European Collection of Authenticated Cell Culture. Following Elmorsy et al. (2023), they were cultured in Dulbecco’s modified Eagle Medium. Initially, SH-SY5Y cells were seeded in 25 cm^2^ flasks covered with poly-d-lysine hydrobromide (5 mg/ml). After the cells reached 60% confluency, they were cultured in a 10% FBS medium. The cells were then grown in SH-SY5Y media containing 10 μM all-trans retinoic acid (RA) and kept out of the light for 3 days to produce cells with a dopaminergic (DA) phenotype. After that, the media was discarded, fresh media containing (80 nM) of 12 O-tetradecanoyl-phorbol- 13-acetate (TPA) was added, and the cells were incubated for 3 days to differentiate. Differentiation was validated by ELISA’s higher levels of tyrosine kinase in the differentiated DA neurons using an Abcam commercial kit.

### Determination of Cytotoxicity and Cellular Viability of SH-SY5Y Cells

The cytotoxic effects of CPF and MPTP on the differentiated cells were assessed using an MTT assay, following the procedures outlined in a prior publication (Elmorsy et al. 2014). A 96-well plate was used for cell subculturing, with 1 × 10^4^ cells/well seeded and an overnight incubation period. They received 72 h of treatment with FXL (1, 2.5, 5, 10, and 20 μM) or CPF and MPTP (0.1, 1, 10, 100, or 1000 μM). After adding MTT reagent to each well, the cells were incubated for two hours. Each well was then filled with 100 μl of solubilizing reagent. A Dyne MRX microplate reader (Dyne Technologies, Chantilly, VA, USA) was used to measure the absorbance at 590 nm after an extra hour of incubation and 30 s of medium-speed shaking. In the experiment, each cell line underwent at least three tests. MTT assay was repeated in the presence and absence of coculture with antioxidant GSH-R (10 μM), caspase- 3 inhibitor z-VAD-fmk (200 μM), or mitochondrial enhancer Co-Q10 (1 μM).

### Bioenergetics Assays

For bioenergetic assays, cells were seeded and incubated till 80–90% confluence, then treated with FXL (2.5 or 5 μM) for 48 h in the presence or absence of CPF or MPTP (50 μM).

### Intracellular ATP Levels Assay

At the time point, the luminous ATP Detection Assay Kit (Abcam, ab113849) quantified intracellular ATP levels using a luminous reaction. Enzymatic conversion of D-luciferin to oxyluciferin by luciferase in the presence of ATP and oxygen produces light emission proportional to ATP concentration. Cells were incubated in opaque white-walled 96-well plates to enhance luminescence signal detection. After experiments, 100 μL of detergent solutions were applied to each well to lyse cells and stabilize ATP. The plate was incubated at room temperature for 5 min. Next, 50 μL of substrate solution with luciferase and luciferin was added to each well and incubated for 5 min. The plate was darkened for 10 min to eliminate background luminescence. A microplate Perkin Elmer “TopCount” luminometer monitored emitted light intensity, which correlated with sample ATP concentration. At least three experiments were performed in triplicate for each treatment concentration.

### Mitochondrial Membrane Potential (MMP) Assay

A 96-well plate was used for cell subculturing, with 1 × 10^4^ cells/well seeded and an overnight incubation period. Subsequently, the cells were treated for 48 h with CPF or MPTP (50 μM) in the presence or absence of FXL (2.5 or 5 μM). The cells underwent treatment with Rh- 123 (50 nM) for a duration of 10 min and were subsequently kept wrapped in aluminum foil at 37 °C. Fluorescence was quantified using excitation and emission wavelengths of 480 nm and 530 nm, respectively.

### Mitochondrial Complexes I (MCI) and III (MCIII) Activities Assay

For mitochondrial complexes, cells were seeded in T25 flasks and incubated till 90% confluence. Then, mitochondrial enriched fractions or cell lysate was obtained for MCI and MCII assays, respectively. Protein content for each sample was measured via Bradford assay following Spinazzi et al. (2012).

Complex I activity was determined by monitoring the oxidation of NADH to NAD^+^ at 340 nm. The assay mixture contained 25 mM potassium phosphate buffer (pH 7.2), 5 mM MgCl₂, 2 mM KCN, 2.5 mg/mL fatty acid-free bovine serum albumin, 130 μM NADH, and 65 μM ubiquinone- 1. The reaction was initiated by adding 10–20 μg of mitochondrial protein, and the decrease in absorbance at 340 nm was recorded for 3–4 min. Rotenone (2 μM) was used to determine rotenone-insensitive activity, which was subtracted from the total activity to yield specific complex I activity.

Complex III activity was measured by following the reduction of cytochrome c at 550 nm. The assay buffer consisted of 25 mM potassium phosphate (pH 7.2), 5 mM MgCl₂, 2 mM KCN, and 50 μM oxidized cytochrome c. The reaction was initiated by adding 50 μM reduced decylubiquinol and 10–20 μg of mitochondrial protein. The increase in absorbance at 550 nm was monitored for 2–3 min. Antimycin A (2 μg/mL) was used to assess antimycin A-insensitive activity, which was subtracted from the total to obtain specific complex III activity.

All assays were performed at 37 °C using a temperature-controlled spectrophotometer. Protein concentrations were determined by the Bradford method to normalize enzyme activities. The complex assay was conducted for at least 6 samples for each treatment for robust data.

### Oxygen Consumption Rate (OCR) Assay

The cells’ OCR was assessed using Clark oxygen electrodes (Rank Brothers Ltd., Newbury, UK). Cells of different samples were suspended in Hank solution with known density (10^4^ − 10^5^) per electrode chamber). The basal OCR of the cells was evaluated for 5 min; then, Na azide (cytochrome oxidase blocker) was added to each chamber. OCR was evaluated via measuring the linear slopes of the oxygen curve for each sample and normalized to the total number of chamber’s cells. At least 6 samples of each treatment concentration data were used for robust data.

### Lactate Production Assay

One typical result of anaerobic glycolysis is the production of lactate. The study looked at how a specific neurotoxin affected DA cells. In 24-well plates, 5 × 10^5^ cells were placed in each well and incubated overnight. Subsequently, the cells were treated for 72 h with FXL (2.5 or 5 μM) in the presence or absence of CPF or MPTP (50 μM). Following the instructions from the manufacturer (Biovision Inc., Milpitas, CA, USA), the media were collected, and lactate levels were measured colorimetrically using a commercial lactate test kit (Catalog Number: K667 - 100). Absorbance was read at 570 nm. Each assay point was carried out three times.

### Mitophagy Proteins Assay

Mitophagy was assessed by studying the levels of PINK1 and PARKIN proteins by a commercial ELISA kit following the manufacturers (Bioscience and Abcam, respectively) using cell lysate. After adding the stop solution, absorbance was read at 450 nm by a Dyne MRX microplate reader. Levels were estimated by substituting OD450 estimated values with the prepared standard curve data.

### Pyruvate Dehydrogenase (PDH) Enzyme Assay

The pyruvate dehydrogenase (PDH) activity colorimetric assay kit (Abcam, ab287837) is used to quantify PDH activity in biological samples. This assay utilizes PDH to convert pyruvate into an intermediate, which subsequently reduces a developer, producing a colorimetric change with high absorbance at 450 nm. For the assay, tissues (10 mg) or cells (1 × 10^6^) should be homogenized in 100 μL of ice-cold PDH Assay Buffer and incubated for 10 min. Following centrifugation at 10,000 × *g* for 5 min, the supernatant is collected, and 5–50 μL of the sample is added per well, with the volume adjusted to 50 μL using PDH Assay Buffer. To assess mitochondrial PDH activity, mitochondria must be isolated from fresh tissues or cells, and 5–50 μL of the mitochondrial sample should be used per well, adjusted to 50 μL with PDH assay buffer. To prepare a PDH Positive Control,10 μL of the provided control is added to the designated well(s), with the final volume adjusted to 50 μL using PDH assay buffer. The reaction is initiated by adding 50 μL of PDH reaction mix to each well, followed by incubation at room temperature for 30 min, protected from light. PDH activity is then measured based on absorbance at 450 nm via an ELISA Dyne MRX microplate reader.

### Alpha-keto Glutamate (α-KG) Assay

Alpha-ketoglutarate (α-KG) concentrations were measured utilizing the alpha-ketoglutarate assay kit (colorimetric) from Abcam (Catalog No. ab83431), in accordance with the manufacturer’s instructions. Initially, samples underwent deproteinization via a 10-kDa molecular weight cutoff spin filter to remove interfering proteins. The assay reaction was initiated by adding 50 μL of each sample or α-KG standard to a 96-well plate. Subsequently, 50 μL of the reaction mix, comprising the α-KG assay buffer, developer, and enzyme mixture, was dispensed into each well. The plate was incubated at 37 °C for 30 min, shielded from light. After incubation, absorbance was quantified at 570 nm utilizing an ELISA Dyne MRX microplate reader.

### Measurements of Oxidative Stress Markers

Cells were seeded and incubated till 80–90% confluence, then treated with FXL (2.5 or 5 μM) for 48 h in the presence or absence of CPF or MPTP (50 μM). Next, the oxidative stress indicators listed below were measured.

### Effect of CPF, MPTP, and FXL on Reactive Oxygen Species (ROS) Production

The experiment followed the protocol outlined by Elmorsy et al. (2014) using dichlorofluorescein diacetate (DCFDA) solution (25 μM in Hank’s solution). Fluorescence readings were taken with excitation/emission wavelengths of 485/535 nm. The experiments were performed in triplicates for each time point.

### Effect of CPF, MPTP, and FXL on Thiobarbituric Acid Reactive Substances (TBARS) Production

A commercial Abcam kit (Catalog Number: ab118970, Abcam, Cambridge, UK) was utilized to quantify the levels of TBARS, specifically malondialdehyde (MDA), in treated homogenized cells. The protocol provided by the manufacturer was followed meticulously. The absorbance was measured at 532 nm. Each experiment was conducted in triplicate.

### Effect of CPF, MPTP, and FXL on Antioxidant Enzymes Activity

The impact of CPF, MPTP, and FXL on the activity of catalase (CAT) and superoxide dismutase (SOD) in DA cells was evaluated. CAT was colorimetrically assessed following the method described by Singh et al. (2008). SOD activity was measured using a colorimetric commercial assay kit (Catalog Number: ab65354, Abcam, Cambridge, UK) as per the provided instructions. The absorbance was measured at 620 nm and 440 nm for CAT and SOD, respectively. Each experiment was performed in triplicate.

### Apoptosis Assays

Cells were subjected to 48 h of exposure to FXL (2.5 or 5 μM) for 48 h in the presence or absence of CPF or MPTP (50 μM) for apoptosis tests. Following the given protocol, the effect of CPF, MPTP, and FXL on DA cell’s caspase 3 (common apoptosis pathway), 8 (extrinsic apoptotic pathway), and 9 (intrinsic apoptosis pathway) activities was then assessed using Flourometric caspase assay kits (Clontech Laboratories Inc., Mountain View, CA, USA). The fluorescence was measured at excitation/emission wavelengths of 400/505, 400/505, and 380/460 nm for caspases − 3, − 8, and − 9, respectively.

### Determination of Gene Expression by Quantitative Polymerase Chain Reaction

Cells were treated with FXL (2.5 or 5 μM) for 48 h in the presence or absence of CPF or MPTP (50 μM) for 48 h. After cell harvesting, RNA was extracted using a commercial kit from Qiagen, Germany. Quantitative PCR reactions were carried out using the CFX96 real-time System from Bio-Rad Laboratories, Inc. The RT^2^ SYBR® Green qPCR Mastermixes kit (Catalog Number: 330513, Qiagen, Hilden, Germany) was used according to the manufacturer's instructions. The primer sequences used are detailed in Table S1. The thermocycling parameters were adjusted as per Martínez et al. (2020). The internal reference housekeeping gene, glyceraldehyde- 3-phosphate dehydrogenase (*GAPDH*), was utilized to quantify and normalize transcript levels. Each experiment was performed in triplicate.

### Bioinformatic Verification

Molecular docking was conducted and visualized via SwissDock to analyze the interactions of CPF, MPTP, and FXL with critical mitochondrial, apoptotic, and oxidative stress-related proteins. Protein structures for ND1, ND5, cytochrome b (cy.b), cytochrome oxidase 1 (Co1), ATP synthase subunits 6 and 8 (ATP 6/8), catalase, superoxide dismutase, Nrf2, HO- 1, caspases 3, 8, and 9, Bax, and BCL2 were sourced from the Protein Data Bank (PDB). Ligand structures were sourced from the PubChem database, with optimized three-dimensional conformations ensured. Docking simulations were performed with SwissDock, during which receptor proteins were prepared by eliminating water molecules and incorporating hydrogen atoms to enhance docking conditions. Ligands underwent energy minimization utilizing the MMFF94 force field prior to docking. Binding affinities, hydrogen bond interactions, hydrophobic interactions, and the involved residues were analyzed utilizing BIOVIA Discovery Studio Visualizer.

### Statistical Analysis

The concentration–response relationships were quantified using non-linear curve fitting statistics, with the top and bottom of the curve constrained to 100 and zero levels, to estimate the MTT EC50 s. A one-way ANOVA was combined with Tukey’s post-test to evaluate data from three or more groups. *P* < 0.05 denotes statistical significance in the statistical analyses carried out with GraphPad Prism 5 (GraphPad Software Inc., San Diego, CA, USA). Multiple variable analyses clustering heatmap and principle component analysis were conducted using Srplot platform for data visualization and graphing.

## Results

### FXL Enhances the Viability of SH-SY5Y Cells Exposed to CPF or MPTP

**Fig. 1:**
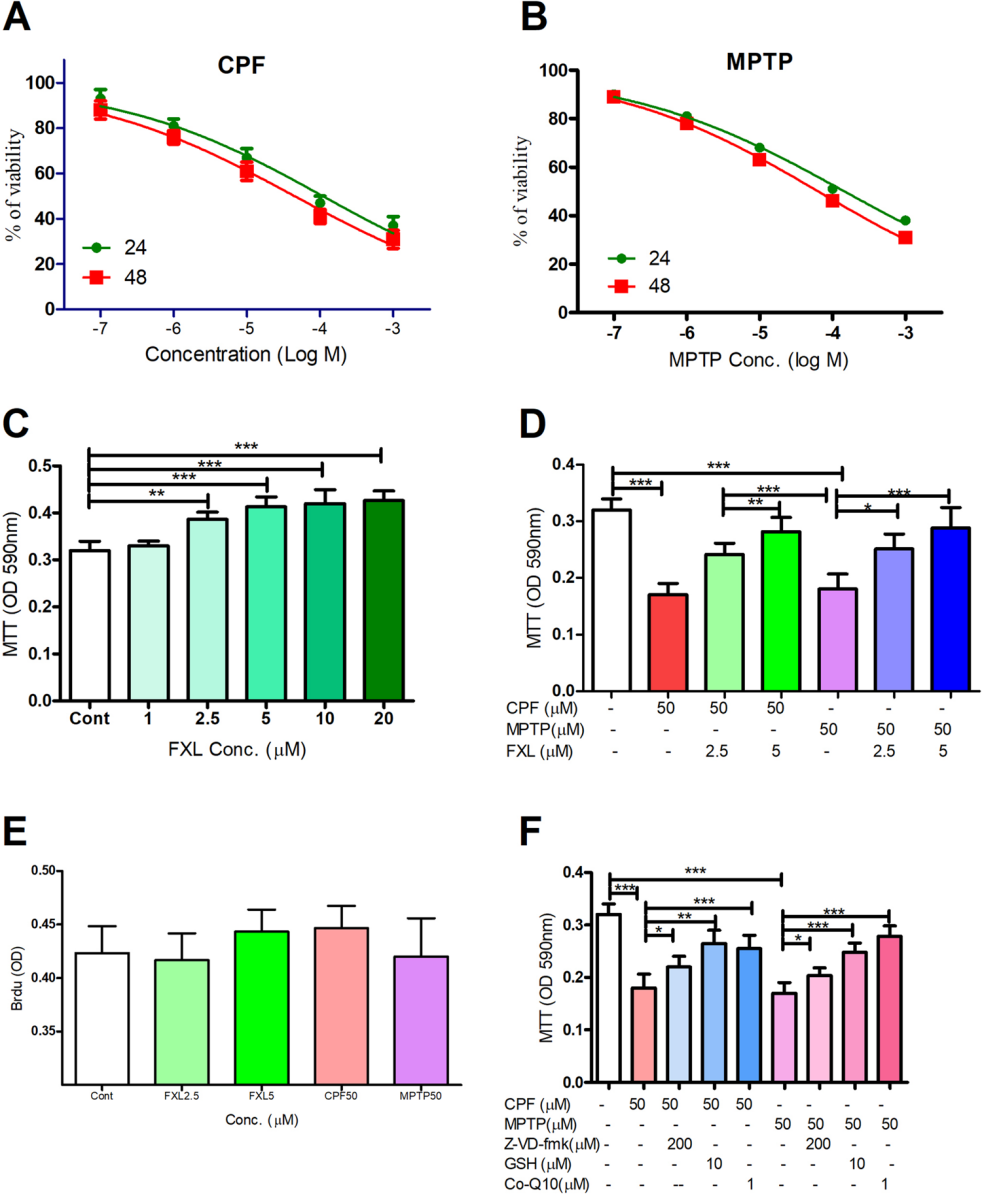
MTT viability assay for the effect of chlorpyrifos (CPF), MPTP, and fucoxanthinol (FXL) on the viability of the dopaminergic differentiated human neuroblastoma cells (SH-SY5Y). Data revealed that CPF (**A**) and MPTP (**B**) decreased the viability of the treated cells in a concentration dependent pattern, while FXL improved the cells viability (**C**). Cotreatment with FXL at concentrations 2.5 and 5 μM significantly mitigated the cytotoxic effect of CPF and MPTP (50 μM) on the treated neuronal cells (**D**). Brdu assay showed that CPF and MPTP (50 μM) and FX (2.5 and 5 μM) have no significant effect on the cells proliferation rates (**E**). Cotreatment with caspase 3 inhibitor, antioxidant, and mitochondrial enhancer significantly mitigated the cytotoxic effect of the pesticides on the treated dopaminergic cells, which suggest roles for apoptosis, oxidative stress, and mitochondrial disruption in the tested pesticide-induced toxic effect. Data was represented as means ± SD. One-way ANOVA with Tuckey’s multiple comparisons post hoc were used to check for the significance (*n* = 9). *P < 0.05, **P < 0.01, *P < 0.001

The cytotoxic effect on the differentiated dopaminergic neurons was assessed using various concentrations. CPF and MPTP were shown to be cytotoxic to the dopaminergic phenotype based on their concentration 24- and 48-h post-exposure (Fig. 1A, B) with an estimated EC50 for CPF of 102 μM (95% confidence interval = 93.5–113.4 μM) and 45.3 (95% confidence interval = 39.5–53.4 μM) following 24 and 48 h post-exposure, respectively. In comparison, estimated EC50 s for MPTP were 153 μM (95% confidence interval = 141.5–159.4 μM) and 54.3 (95% confidence interval = 47.5–61.3 μM) following 24 and 48 h post-exposure, respectively. FXL at 2.5, 5, 10, and 20 μM concentrations significantly increased the differentiated dopaminergic cells’ viability to variable extents without significant difference among the effect of 1 μM concentration. For example, FXL at 2.5 and 5 μM increased, the tested cells’ viability to 118.9 ± 3.2 and 130.2 ± 3.5% of the controls’ viability, respectively (Fig. 1C). Interestingly, cotreatment with submaximal concentrations of FXL (2.5 or 10 μM) alongside CPF or MPTP (50 μM) significantly alleviated pesticide-induced cytotoxicity in dopaminergic (DA) cells. (Fig. 1D) to variable extents. We also tested the effect of FXL (2.5 and 5 μM), CPF (50 μM), and MPTP (50 μM) on the cells’ proliferation by BrdU assay. Data revealed that FXL and pesticides did not affect the treated cells’ proliferation (Fig. 1E).

The impact of anti-caspase- 3, antioxidant, and mitochondrial enhancers was evaluated to further investigate the pesticide-induced cytotoxic effect. In CPF cytotoxicity, cotreatment with anti-caspase, antioxidant, or mitochondrial enhancer improved the viability by approximately 23.2 ± 3.2, 43.5 ± 3.3, and 39.2 ± 3.5%, respectively, while in MPTP cytotoxicity, the cotreatments improved the viability by about 18.3 ± 3.4, 39.5 ± 3.3, and 52.4 ± 3.2, respectively (Fig. 1F).

### FXL Restores PDH and ATP and Mitophagy Protein Levels in SH-SY5Y Cells Exposed to CPF or MPTP

**Fig. 2:**
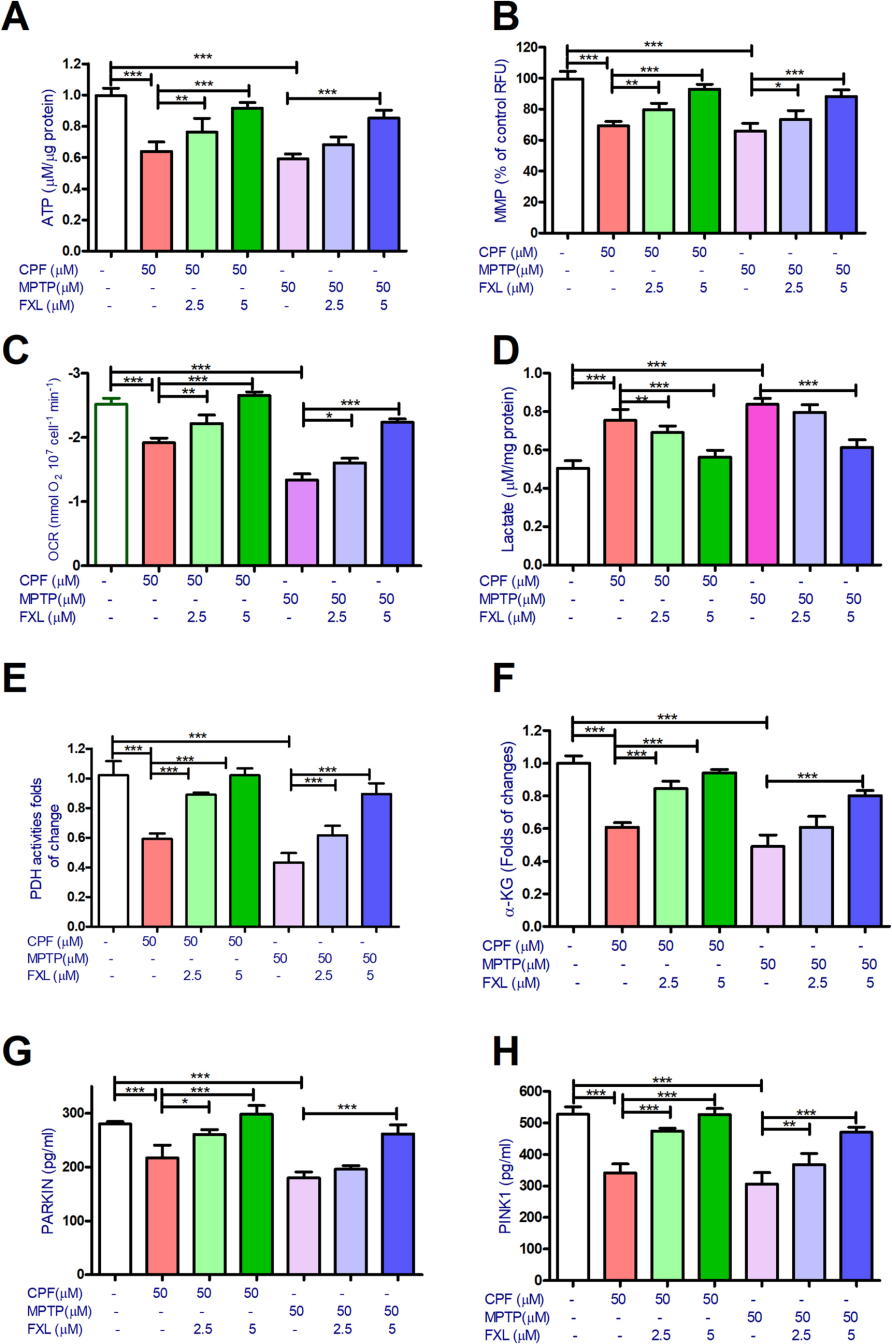
The effect of chlorpyrifos (CPF), MPTP, and fucoxanthinol (FXL) on the mitochondrial bioenergetics parameters of the dopaminergic differentiated human neuroblastoma cells (SH-SY5Y). Data revealed that CPF (50 μM) and (50 μM) decreased ATP production (**A**), mitochondrial membrane potential (MMP) (**B**), oxygen consumption rates (OCRs) (**C**), with increased lactate production levels (**D**). In addition the tested pesticides were shown to decrease the levels of pyruvate kinase activities (PKA) (**E**), alpha-keto glutarate (α-KG) (**F**), mitophagy proteins Parkin (**G**), and PIINK1 (**H**). Cotreatment with FXL at concentrations 2.5 and 5 μM significantly mitigated the effect of CPF and MPTP (50 μM) on the bioenergetics parameters of treated neuronal cells (**A**–**H**). Data was represented as means ± SD. One-way ANOVA with Tuckey’s multiple comparisons post hoc were used to check for the significance (*n* = 9). *P < 0.05, **P < 0.01, ***P < 0.001

Bioenergetic assays revealed that CPF and MPTP dramatically reduced ATP levels to 63.3 ± 3.7 and 57.7 ± 2.2% of the control levels. FXL (2.5 or 5 μM) cotreatment significantly alleviated the CPF effect on ATP production, while only FXL (5 μM) showed a significant protective effect with MPTP-treated cells. FXL (5 μM) cotreatment improved ATP levels to 90.7 ± 3.2 and 83.6 ± 3.5% of the control levels (Fig. 2A). The MMP assay showed data similar to that of the ATP assay. CPF and MPTP were found to dramatically reduce ATP levels to 68.9 ± 3.4 and 64.2 ± 3% of the control levels. FXL (2.5 or 5 μM) cotreatment significantly alleviated the CPF effect on MMP production to variable extents. FXL (5 μM) cotreatment improved MMP levels to 92.7 ± 3.5 and 87.5 ± 3.1% of the control levels (Fig. 2B).

OCR assay showed that CPF and MPTP dramatically reduced ATP levels to 78.2 ± 3.2 and 54.2 ± 3.5% of the control levels. FXL (2.5 or 5 μM) cotreatment significantly alleviated the CPF effect on MMP production to variable extents. FXL (5 μM) cotreatment improved MMP levels to 104.2 ± 2.5 and 86.7 ± 2.2% of the control levels (Fig. 2C). In parallel, lactate assay in the media supernatant showed that CPF and MPTP dramatically increased media lactate levels to 152.2 ± 3.1 and 165.7 ± 2.8% of the control levels. FXL (5 μM) cotreatment significantly alleviated the CPF effect on MMP production to 112.3 ± 3.2 and 104.3 ± 2.6% of the control media lactate levels (Fig. 2D). In addition, CPF and MPTP were found to significantly decrease PDH activity levels to 58.7 ± 2.2 and 43.7 ± 3.4% of the control levels and decrease α-KG of the treated cells to 62.3 ± 2.2 and 48.9 ± 3.6% of the control levels, respectively. FXL (2.5 or 5 μM) cotreatment with the pesticide significantly alleviated the CPF and MPTP effects on the MMP of the treated cells (Fig. 2E and F). The impact of pesticides on the mitophagy proteins; PARKIN and PINK1 proteins was assessed using an ELISA kit to determine mitophagy. Data showed that CPF and MPTP significantly decreased PARKIN levels to 75.4 ± 4.2 and 64.6 ± 3.7% of the control levels and decreased PINK1 of the treated cells to 65.6 ± 2.7 and 56.7 ± 3.5% of the control levels, respectively. FXL (2.5 or 5 μM) cotreatment with the pesticide significantly alleviated the CPF effect on MMP effect on mitophagy proteins’ levels in the pesticide-treated cells (Fig. 2G and H).

### FXL Restores the Levels of Mitochondrial Complexes and Antioxidant Enzymes in SH-SY5Y Cells Exposed to CPF or MPTP

**Fig. 3:**
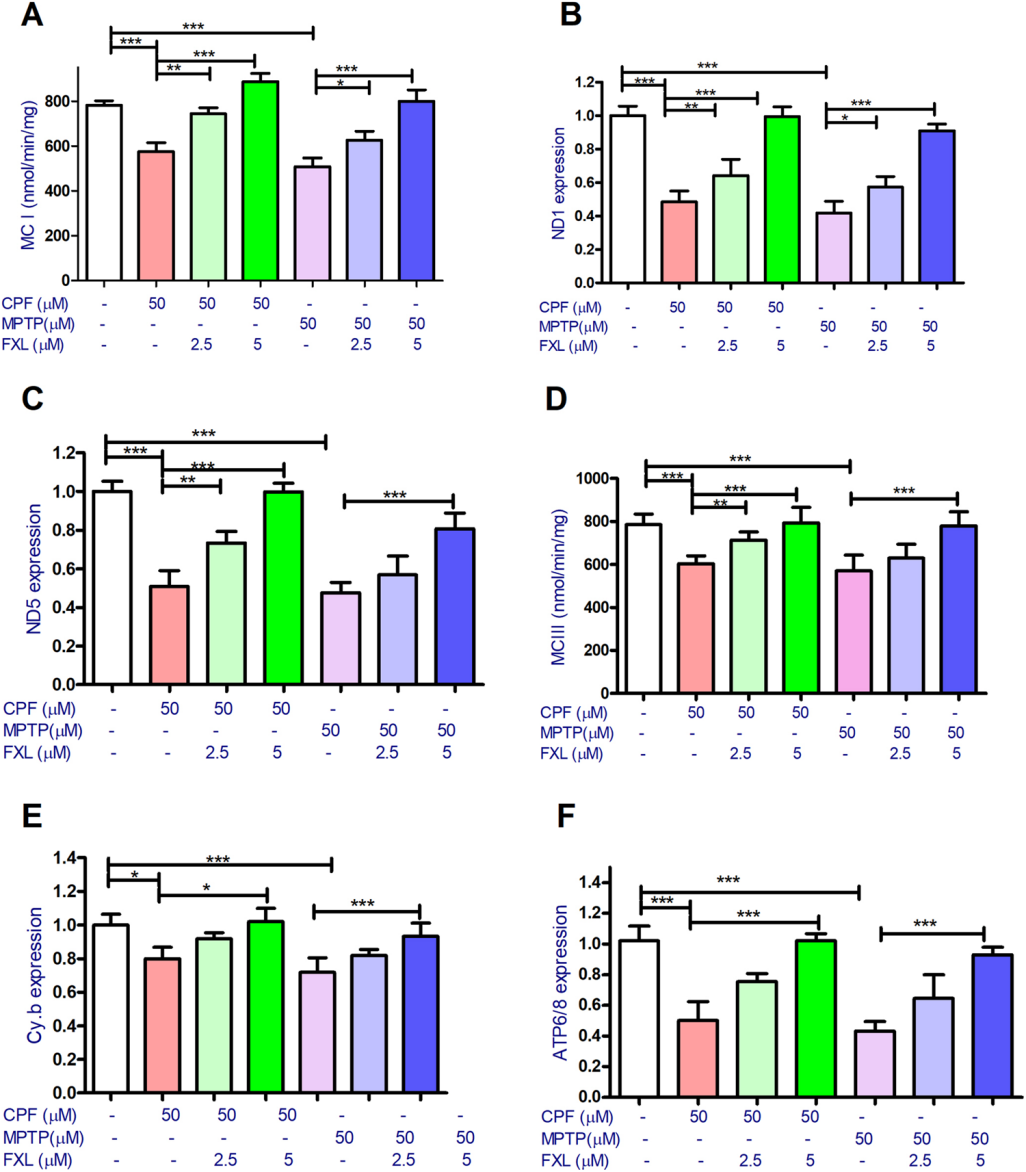
The effect of chlorpyrifos (CPF), MPTP, and fucoxanthinol (FXL) on the mitochondrial complexes (MCs) of the dopaminergic differentiated human neuroblastoma cells (SH-SY5Y). Data revealed that CPF (50 μM) and (50 μM) decreased the activity of MCI (**A**), and its subunits coding genes ND1 (**B**), ND5 (**C**). Also, the tested pesticides were shown to decrease the activity of MCIII (**D**), and its coding genes cyt.b (**E**), as well as the MC IV subunits 6 and 8 ATP6/8 (**F**). Cotreatment with FXL at concentrations 2.5 and 5 μM significantly mitigated the effect of CPF and MPTP (50 μM) on the tested MCs and their coding genes in the treated neuronal cells (**A**–**H**). Data was represented as means ± SD. One-way ANOVA with Tuckey’s multiple comparisons post hoc were used to check for the significance (*n* = 9). *P < 0.05, **P < 0.01, ***P < 0.001

The effect of pesticides and FXL on MCI and MCIII was evaluated. Both pesticides significantly decreased the activity of MCI and MCIII and their studied coding gene expression. FXL (2.5 and 5 μM) cotreatment was found to counteract the CPF and MPTP effect on MCI, MCIII, and the coding genes to variable extents based on its concentration (Fig. 3A–E). For *ATP 6/8* gene expression data, pesticides significantly decreased its expression with a protective effect for FXL (2.5 and 5 μM) cotreatment (Fig. 3F).

**Fig. 4:**
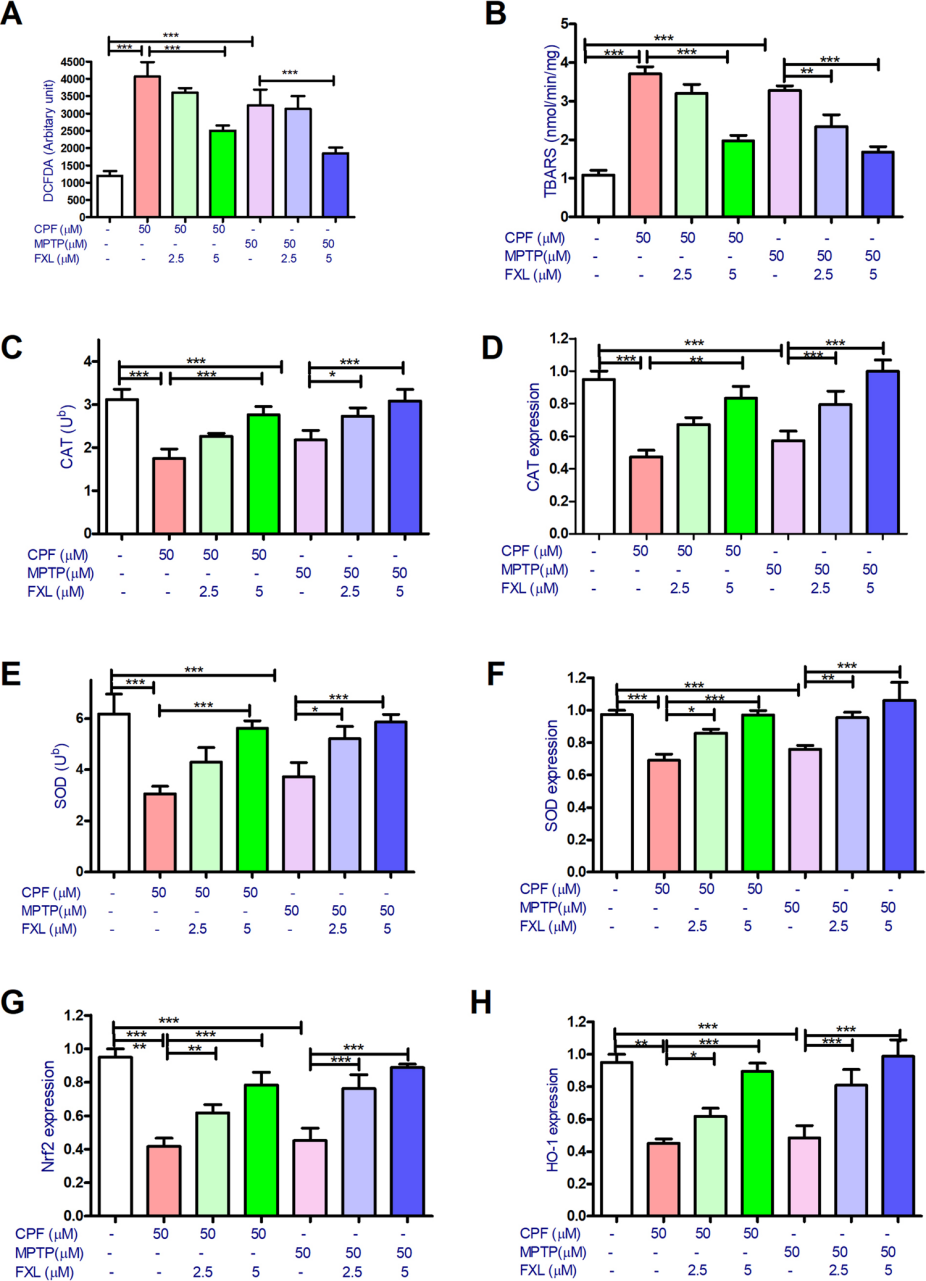
The effect of chlorpyrifos (CPF), MPTP, and fucoxanthinol (FXL) on oxidative stress parameters of the dopaminergic differentiated human neuroblastoma cells (SH-SY5Y). Data revealed that CPF (50 μM) and (50 μM) significantly increased reactive oxygen species (ROS) production (**A**) and lipid peroxidation with increased thiobarbituric acid (TBARS) byproduct in the treated cells (**B**). Also, both pesticides decreased the activity of antioxidants enzymes catalase (CAT) (**C**) and its coding gene (**D**) and superoxide dismutase (SOD) (**E**) and its coding subunit gene SOD1 (4 F), in addition to the expression of the antioxidant genes Nrf2 (G), and HO- 1 (**H**). Cotreatment with FXL at concentrations 2.5 and 5 μM significantly mitigated the effect of CPF and MPTP (50 μM) on the tested oxidative stress parameters in the treated neuronal cells (**A**–**H**). Data was represented as means ± SD. One-way ANOVA with Tuckey’s multiple comparisons post hoc were used to check for the significance (*n* = 9). *P < 0.05, **P < 0.01, ***P < 0.001

Regarding the effect of neurotoxic pesticides on oxidative stress markers. CPF and MPTP (50 μM) significantly increased ROS levels to around 3.3- and 2.7-folds and lipid peroxidation estimated levels to 3.7- and 3.3-folds of the basal condition, respectively. While FXL (2.5 and 5 μM) cotreatment with the pesticides significantly alleviated their effect on ROS levels and lipid peroxidation to variable extents (Fig. 4A and B).

Regarding the impact of the pesticides and FXL cotreatment on antioxidant enzymes CAT and SOD and their coding genes expression, CPF was found to decrease CAT activities and its gene expression to 56.3 ± 2.1 and 45.7 ± 1.8% of the control levels, respectively. Also, MPTP was found to decrease CAT activities and its gene expression to 73.2 ± 2.3 and 57.8 ± 2.2% of the control levels, respectively. Similarly, SOD assays showed that CPF was found to decrease SOD activities and its gene expression to 61.3 ± 2.9 and 69.7 ± 1.7% of the control levels, respectively. Also, MPTP was found to decrease SOD activities and its gene expression to 69.8 ± 2.5 and 78.8 ± 2.1% of the control levels, respectively. FXL cotreatment with CPF or MPTP was found to significantly alleviate their effect on CAT and SOD activities and coding genes expression (Fig. 4C–F). Regarding *Nrf2* and *Ho- 1* gene expression assays, pesticides significantly inhibited the expression of both genes. This inhibitory action was counteracted by FXL cotreatment (Fig. 4G–H).

### FXL Decreases the Level of Cell Death Markers in SH-SY5Y Cells Exposed to CPF or MPTP

**Fig. 5:**
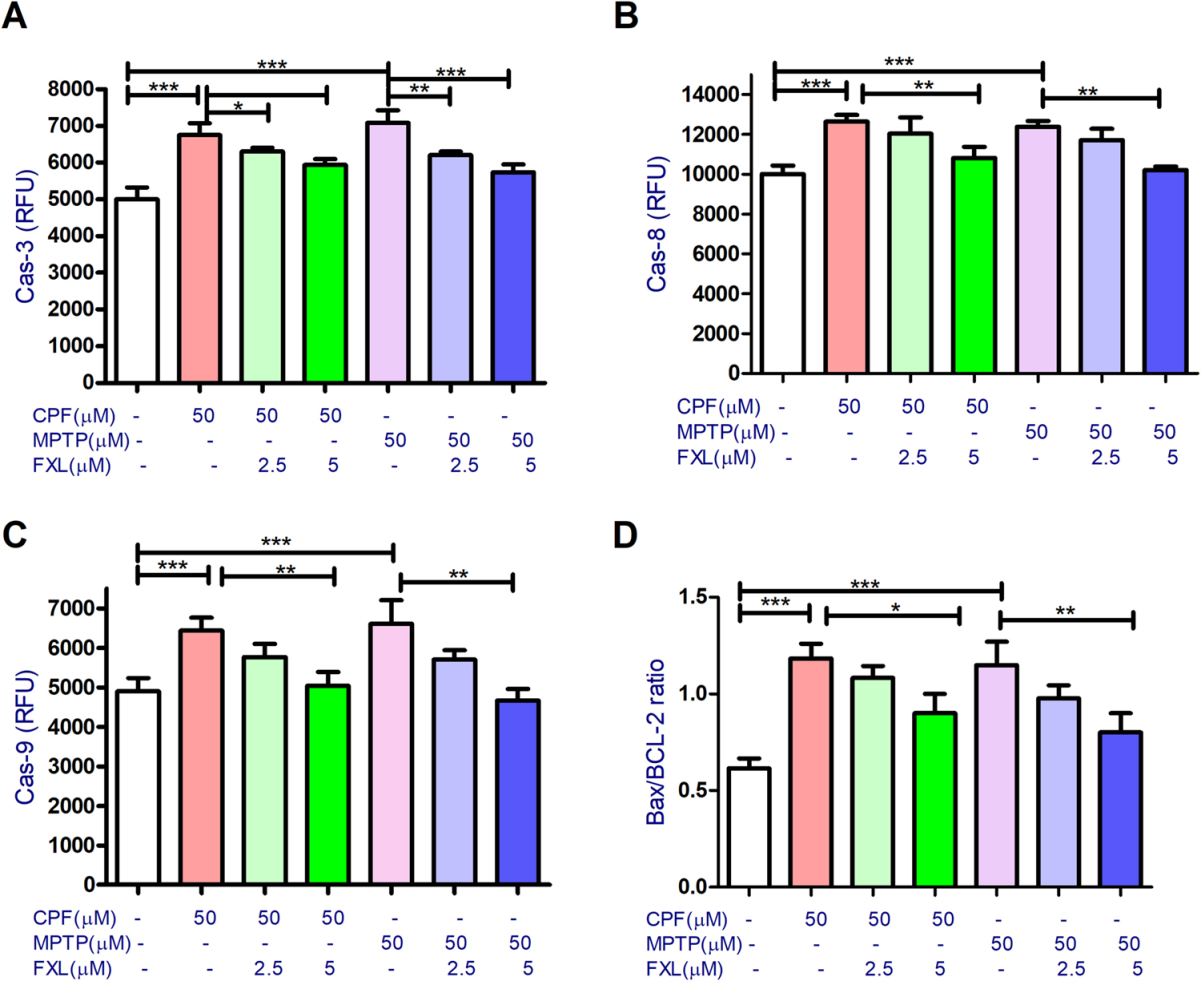
The effect of chlorpyrifos (CPF), MPTP, and fucoxanthinol (FXL) on apoptosis parameters of the dopaminergic differentiated human neuroblastoma cells (SH-SY5Y). Data revealed that CPF (50 μM) and (50 μM) significantly increased the activities of caspases- 3 (**A**), − 9 (**B**), and − 8 (**C**) for variable extents with increased Bax/Bcl2 ratio (**D**) in the treated neuronal cells (**B**). Cotreatment with FXL at concentrations 2.5 and 5 μM significantly mitigated the effect of CPF and MPTP (50 μM) on the tested apoptosis markers in the treated neuronal cells (**A**–**H**). Data was represented as means ± SD. One-way ANOVA with Tuckey’s multiple comparisons post hoc were used to check for the significance (*n* = 9). *P < 0.05, **P < 0.01, ***P < 0.0001

Caspases assays revealed that both pesticides (50 μM) significantly (*P* < 0.0001) increased caspases − 3, − 8, and − 9 levels in comparison to the non-treated cells (Fig. 5A–C), with a concomitant increase in Bax/Bcl2 ratio in contrast to the controls’ levels (Fig. 5D). FXL cotreatments significantly antagonize the effect of pesticides on caspases and apoptosis-related gene expression for variable extents (Fig. 5A–D).

The bioinformatics data showed that CPF mainly triggered the pathways of apoptosis (Bcl2, Bax, and CASP3), oxidative stress (BCHE) as well as the inflammatory response (TNF and IL1β) in addition to cholinesterase genes affection (ACHE and BCHE) (Figure S1). MPTP was shown to interact with genes related to apoptosis, inflammatory response, and nitric oxide synthase (NOS) genes which code for NOS in the mitochondrial outer membrane with proved role in oxidative damage and apoptosis. Also, MPTP was found to interact with other genes related to neurotransmitter synthesis (TH), dopamine transporters (*SLC6 A3*), alpha-synuclein synthesis (SNCA), and structural filament proteins of the mature astrocytes (MAOB) (Figure S2). While there was no specific data for FXL, its parent flavonoid fucoxanthin was found to exert its protective effect via interaction with pathways of inflammatory response (IL6, PTGS2, and TNF). Tumor necrosis factor-alpha (TNF-α) is a key pro-inflammatory cytokine that plays a central role in neuroinflammation and neuronal apoptosis and oxidative stress (Zahedipour et al. 2022). Also, Fucoxanthin was found to affect apoptosis pathways via interaction with Bcl2L1 and Bcl2. B-cell lymphoma 2 (Bcl- 2) is an anti-apoptotic protein that preserves mitochondrial integrity and prevents cytochrome c release, thereby inhibiting caspase-dependent apoptosis (Zeng et al. 2018). Also, bioinformatics data showed that fucoxanthin can interact with oxidative damage and DNA repair (NOS2 and PARP1) and bioenergetics (ADCY7). Inducible nitric oxide synthase (NOS2) is a key enzyme involved in neuroinflammation and oxidative stress, contributing to neuronal damage by producing excessive nitric oxide (NO) (Yadav et al. 2017). In addition, fucoxanthin was shown to interact with Akt1 which is essential for AKT/PI3 K signaling pathways involved in various cellular biological processes (Figure S3).

**Fig. 6:**
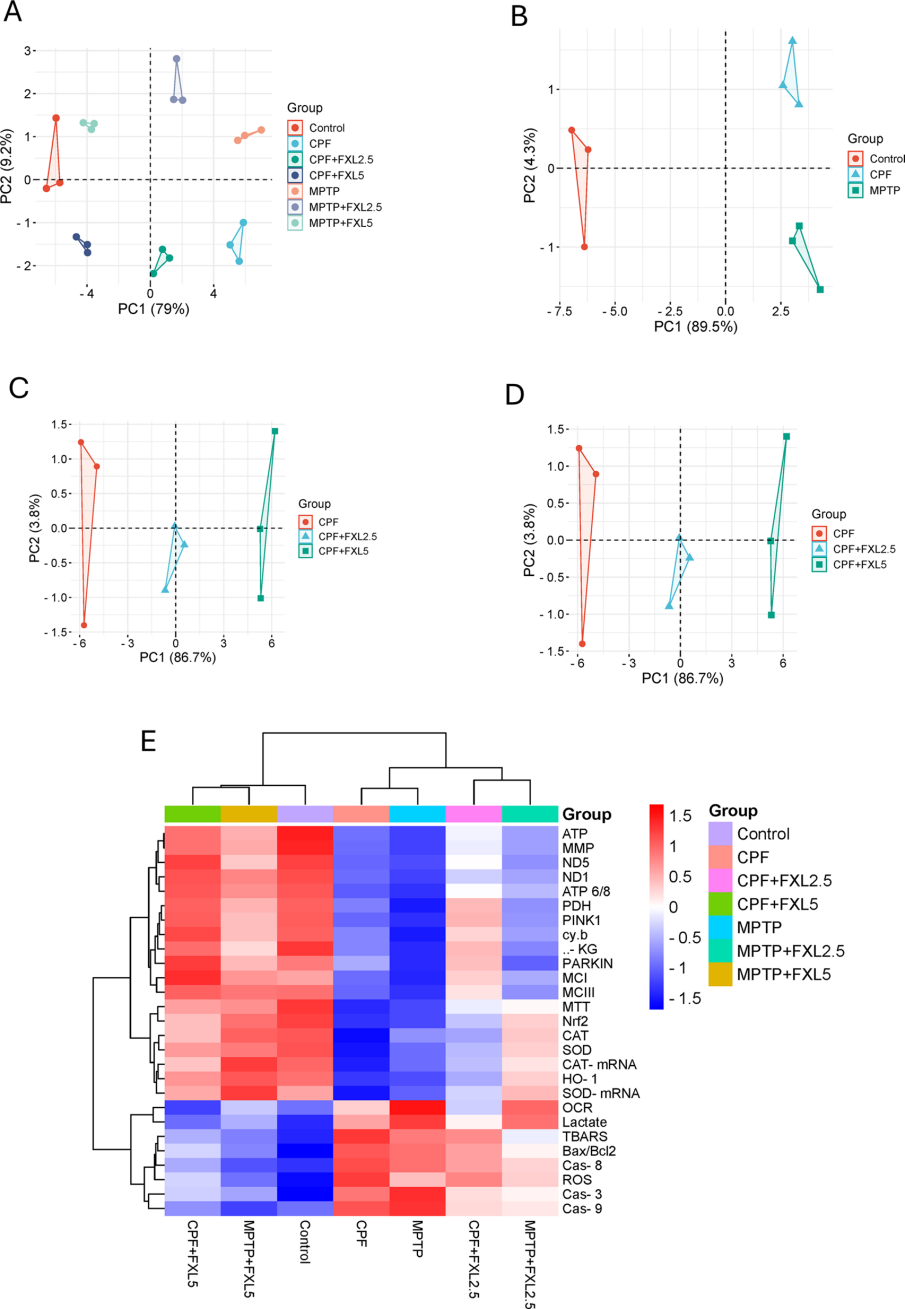
Multivariable data analysis of the in vitro studies data. The figure illustrates the effects of FXL treatment against CPF- and MPTP-induced neurotoxicity through PCA plots (**A**–**D**) and a clustering heatmap (**E**)

Multivariable analysis was performed using PCA and clustering heatmapping for additional data analysis. The PCA plots (Fig. 6A and B) illustrate a distinct separation among control, toxin-treated, and FXL-treated groups, with CPF and MPTP groups clustering separately from the control group, indicating their significant neurotoxic effects. FXL treatment at both 2.5 and 5 μM results in a notable shift of these clusters towards the control, demonstrating a significant restorative effect. The 5 μM concentration of FXL demonstrates a more pronounced normalization effect, especially in the MPTP-treated group (Fig. 6C and D), indicating a dose-dependent protective response. The spatial separation highlights FXL’s capacity to mitigate the biochemical and molecular disruptions induced by CPF and MPTP exposure, indicating its potential as a neuroprotective agent. The clustering heatmap (Fig. 6E) enhances these findings by offering a detailed visualization of molecular markers associated with oxidative stress, mitochondrial dysfunction, and apoptosis. CPF and MPTP treatments markedly influence the expression of critical biomarkers, characterized by reduced mitochondrial parameters (ATP, MMP, ND5, and cytochrome b), increased oxidative stress (ROS, TBARS), and the activation of apoptosis pathways (caspase- 3, − 8, − 9, Bax/Bcl2). Distinct clusters associated with toxin-treated groups indicate these perturbations. FXL treatment reverses these alterations, evidenced by the normalization of antioxidant markers (Nrf2, CAT, SOD), restoration of mitochondrial function, and inhibition of apoptotic markers. The heatmap indicates that the higher dose of FXL (5 μM) exhibits a more significant effect in restoring these parameters to levels comparable to the control. The PCA and heatmap collectively demonstrate FXL’s capacity to reduce oxidative stress, maintain mitochondrial integrity, and inhibit apoptosis, underscoring its therapeutic potential against CPF- and MPTP-induced neurotoxicity in a dose-dependent manner.

**Fig. 7:**
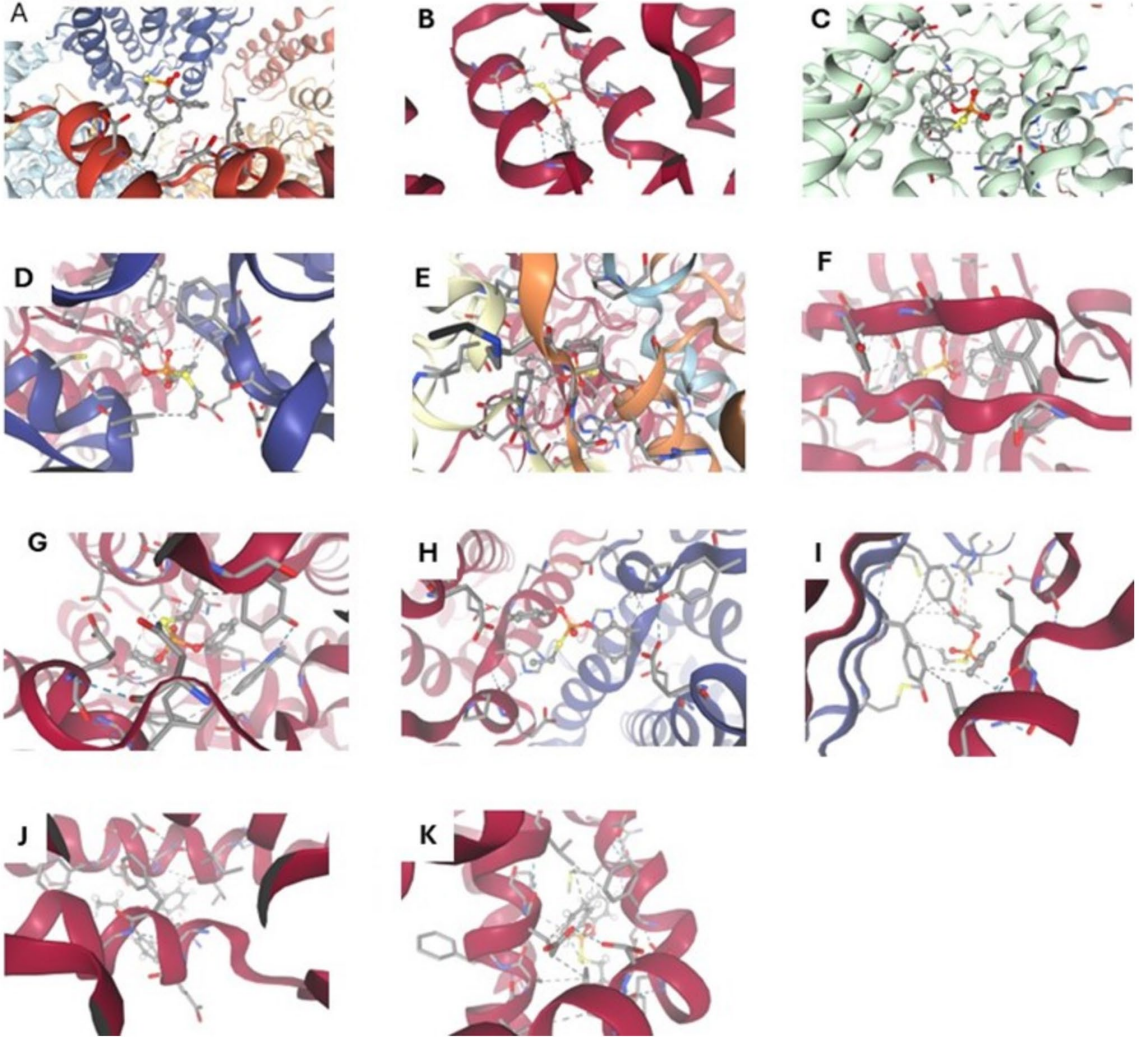
Molecular docking interaction of chlorpyrifos (CPF) with ND1 (**A**), ND5 (**B**), Cy.b (**C**), ATP6/8 (**D**), CAT (**E**), SOD (**F**), Nrf2 (**G**), HO- 1 (**H**), Cas- 3 (**I**), Bax (**J**), Bcl2 (**K**). Hydrogen bonds are represented by blue lines, ionic interactions bonds are represented by yellow lines, cation-π interactions are represented by orange lines, hydrophobic contacts are represented by gray lines, while π-stacking interactions are represented by green lines

**Fig. 8:**
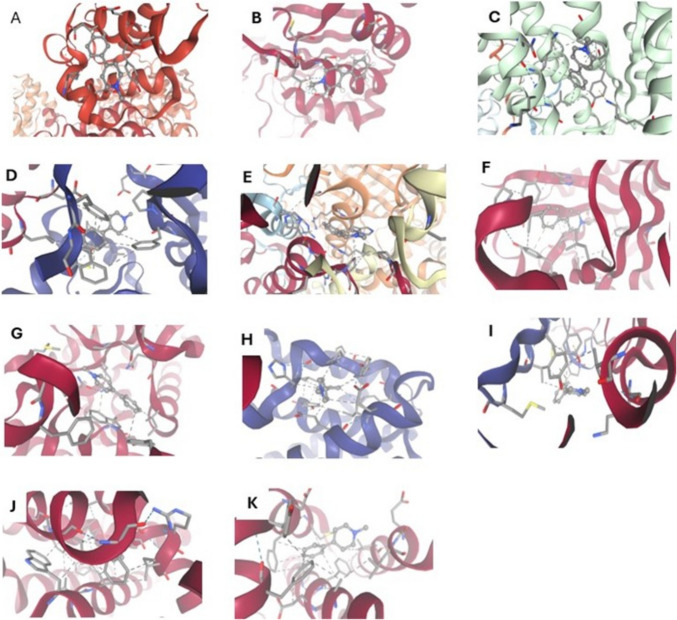
Molecular docking interaction of MPTP with ND1 (**A**), ND5 (**B**), Cy.b (**C**), ATP6/8 (**D**), CAT (**E**), SOD (**F**), Nrf2 (**G**), HO- 1 (**H**), Cas- 3 (**I**), Bax (**J**), Bcl2 (**K**). Hydrogen bonds are represented by blue lines, ionic interactions bonds are represented by yellow lines, cation-π interactions are represented by orange lines, hydrophobic contacts are represented by gray lines, while π-stacking interactions are represented by green lines

**Fig. 9:**
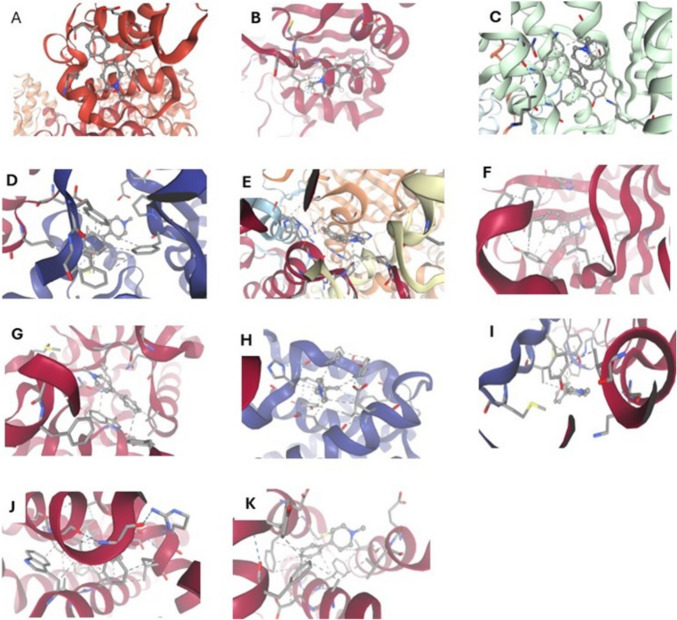
Molecular docking interaction of fucoxanthinol (FXL) with ND1 (**A**), ND5 (**B**), Cy.b (C), ATP6/8 (**D**), CAT (**E**), SOD (**F**), Nrf2 (**G**), HO- 1 (**H**), Cas- 3 (**I**), Bax (**J**), Bcl2 (**K**). Hydrogen bonds are represented by blue lines, ionic interactions bonds are represented by yellow lines, cation-π interactions are represented by orange lines, hydrophobic contacts are represented by gray lines, while π-stacking interactions are represented by green lines

The molecular docking results indicated varying binding affinities of CPF, MPTP, and FXL to the targeted proteins. FXL demonstrated the highest binding affinity with Nrf2 (− 7.8 kcal/mol), catalase (− 7.2 kcal/mol), and BCL2 (− 7.0 kcal/mol), establishing stable hydrogen bonds and notable hydrophobic interactions. The key residues identified are ARG135, GLY259, and TYR67 for Nrf2; HIS74 and TYR72 for catalase; and GLU152 and LEU158 for BCL2. Chlorpyrifos and MPTP exhibited moderate to weak binding affinities, demonstrating minimal interactions at key residues, which suggests reduced efficacy in pathway modulation. FXL exhibited significant binding affinities with mitochondrial proteins, including ND1 (− 6.5 kcal/mol), cytochrome b (− 6.8 kcal/mol), and ATP synthase subunits. Furthermore, interactions with genes associated with apoptosis and oxidative stress, including HO- 1 (− 7.4 kcal/mol) and Bax (− 7.1 kcal/mol), highlighted its capacity to effectively modulate these pathways. CPF and MPTP exhibited lower interaction profiles, with binding energies not surpassing − 5.4 kcal/mol for these proteins. The data collectively indicate the therapeutic potential of FXL and the minimal effects of CPF and MPTP on these pathways (Table 1, Figs. 7, 8, and 9).

**Table 1:** Summary of the molecular docking outcomes regarding interactions of chlorpyrifos (CPF), MPTP, and fucoxanthin (FXL) with genes and proteins related to the mitochondrial functions, oxidative stress and apoptosis

Ligand	Protein/Gene	Affinity (kcal/mol)	Residues involved	Hydrogen bond interactions	Hydrophobic interactions
FXL	Nrf2	− 7.8	ARG135, GLY259, TYR67	ARG135•H•TYR67	TYR67, PHE121
	Catalase	− 7.2	HIS74, TYR72	HIS74•H•TYR72	MET120, VAL125
	BCL2	− 7.0	GLU152, LEU158	GLU152•H•LEU158	ILE154, PHE161
	ND1	− 6.5	SER87, PHE94	SER87•H•PHE94	TRP92, LEU97
	ND5	− 6.7	GLY112, ARG130	GLY112•H•ARG130	PHE129, MET133
	Cytochrome b	− 6.8	ASP67, GLN102	ASP67•H•GLN102	LEU100, PHE105
	Co1	− 7.1	GLN165, LYS172	GLN165•H•LYS172	TYR169, VAL177
	HO- 1	− 7.4	GLU53, HIS78	GLU53•H•HIS78	PHE76, TRP80
	Bax	− 7.1	TYR108, ARG112	TYR108•H•ARG112	LEU110, VAL115
Chlorpyrifos	Nrf2	− 5.2	ARG135, SER200	ARG135•H•SER200	TYR67
	Catalase	− 5.0	HIS74, GLU87	HIS74•H•GLU87	MET120
	BCL2	− 5.4	GLU152, SER195	GLU152•H•SER195	LEU158
	ND1	− 5.1	GLY89, ASP92	GLY89•H•ASP92	VAL95
	ND5	− 5.3	ARG130, PHE133	ARG130•H•PHE133	MET133
	Cytochrome b	− 5.3	GLU55, LEU77	GLU55•H•LEU77	VAL74
	Co1	− 5.2	ASP112, TYR117	ASP112•H•TYR117	LEU115
	HO- 1	− 5.4	GLU53, TYR66	GLU53•H•TYR66	PHE76
	Bax	− 5.1	TYR108, VAL112	TYR108•H•VAL112	LEU110
MPTP	Nrf2	− 5.5	ARG135, SER200	ARG135•H•SER200	TYR67
	Catalase	− 5.3	HIS74, GLU87	HIS74•H•GLU87	MET120
	BCL2	− 5.4	GLU152, SER195	GLU152•H•SER195	LEU158
	ND1	− 5.4	GLY89, ASP92	GLY89•H•ASP92	VAL95
	ND5	− 5.3	ARG130, PHE133	ARG130•H•PHE133	MET133
	Cytochrome b	− 5.4	GLU55, LEU77	GLU55•H•LEU77	VAL74
	Co1	− 5.2	ASP112, TYR117	ASP112•H•TYR117	LEU115
	HO- 1	− 5.5	GLU53, TYR66	GLU53•H•TYR66	PHE76
	Bax	− 5.2	TYR108, VAL112	TYR108•H•VAL112	LEU110

## Discussion

This study evaluated the neuroprotective effect of FXL on CPF and MPTP neurotoxicity using dopaminergic phenotype differentiated SH-SY5Y cells as an in vitro model. The current study utilized several assays to detect cytotoxicity, mitochondrial bioenergetics, oxidative damage, and apoptotic pathways evaluation. The data demonstrate a protective effect of FXL against CPF and MPTP neurotoxicity. It also improved cell viability and decreased the levels of mitophagy proteins, as well as the activity of the PDH enzyme, ROS release, and lipid peroxidation. Concurrently, FXL increased the levels of antioxidant defenses. Multivariable data analysis highlights the neuroprotective effect of FXL to mitigate the cytotoxic effect of CPF and MPTP. In addition, the molecular docking data gives better explanation of the study outcomes and strengths the robustness of the study findings.

Differentiated SH-SY5Y cells are widely used models to study neurotoxicants and their neurotoxicity mechanisms (Presgraves et al. 2004; Schneider et al. 2011). Recent research has demonstrated that differentiated SH-SY5Y cells’ bioenergetic profile shares several characteristics with neurons, such as a bioenergetic reserve capacity (Schneider et al. 2011). These cells have developed into differentiated forms with functional dopamine transporters. It is comparable to synaptosomes despite having less activity than mesencephalic DA neurons (Presgraves et al. 2004; Wernicke et al. 2010). It is critical to understand that different cell types and the expression levels of neurotransmitter receptors, transporters, and dopamine-active transporter (DAT) might affect the relative efficacy of neurotoxicants. Regarding cellular characteristics, primary DA neurons from rodents might be more similar to those from humans. The use of mesencephalic neurons, which are complex and heterogeneous to get in large quantities, is technically limited by the requirement for homogeneous cell populations for cellular bioenergetic study (Cantu et al. 2009).

Differentiation has been widely employed as a model for PD, even though it might not yield pure DA cells (Xicoy et al. 2017). Due to their malignant beginnings, SH-SY5Y cells exhibit genetic abnormalities, yet many genes and pathways that are dysregulated in the development of PD remain intact (Krishna et al. 2014). Most of the nigrostriatal pathway had deteriorated at the initial diagnosis of PD (Hancock et al. 2008), and a significant portion of the remaining nigral neurons was stressed (Bernheimer et al. 1973).

It was crucial to use toxic chemical concentrations in an in vitro model that resulted in roughly 50% cell death, which is comparable to what is anticipated in individuals receiving a diagnosis of PD. This is despite PD being a chronic condition and the toxicity being acute. Results from the MTT assay showed that CPF and MPTP treatment-induced cytotoxicity. FXL was found to have cytoprotective effects and improved cell viability. Previous studies have shown that FX has a potential protective effect on different organ cell lines, including HepG2 liver cells (Zeng et al. 2018), Vero kidney cells (Heo et al. 2008), retinal cells (Liu et al. 2016), and skin cells (Urikura et al. 2011).

The study then evaluated the role of bioenergetic disruption, oxidative damage, and apoptosis in pesticide-induced cytotoxicity using anti-caspase- 3, antioxidant, and mitochondrial enhancers. Data showed the expected role in the three mechanisms of pesticide-induced cytotoxicity. Also, apoptosis was shown to have a small contribution to the outcomes. While oxidative stress was expected to have a significant role in CPF cytotoxicity, mitochondrial disruption was expected to contribute to MPTP cytotoxicity. Then, the three mechanisms were evaluated by further assays.

The mitochondria serve as the primary sites for cellular respiration and oxidative phosphorylation, which produces adenosine triphosphate (ATP) through the Krebs cycle and the electron transport chain containing MCI-IV, which are essential to creating the proton electrochemical gradient via protons pumping in the intermembrane space which is required for ATP production (Brand and Nicholls 2011). In this valuable chain of complexes, mitochondrial complexes I and III are frequently the target of poisons. Furthermore, they often serve as a location for proton leaks and produce dangerous ROS (Sousa et al. 2018). Mitochondrial dysfunction was widely discussed as an underlying mechanism for dopaminergic neuron loss in PD (Haddad and Nakamura 2015). Our study demonstrates that exposure to CPF or MPTP can reduce ATP levels, indicating an impact that results in mitochondrial dysfunction. Furthermore, pesticides were shown to lower MMP and OCR. This was associated with decreased levels of mitophagy proteins PARKIN and PINK1 and reduced activity of the PDH enzyme, the main entry for the Krebs cycle, with a reduced level of α-KG, an intermediate of the cycles. This was shown to occur in parallel with incased lactate release, which is expected with increased anaerobic glycolysis as a source of ATP for an inhibited Krebs cycle. In parallel, assays showed that CPF and MPTP significantly inhibited MCI and MCIII activities in addition to their coding genes *ND1*, *ND5*, and *cyt-b* expression and ATPase subunit 6 and 8 genes expression. Interestingly, FXL significantly alleviated all pesticides'adverse effects on the bioenergetics parameters assays.

The information points to a possible connection between cell death and the elevation of ROS generation in the mitochondria caused by pesticide inhibition of MCI and MCIII with electron leakage and the formation of ROS. It is shown that MCI blockade-related decreased mitochondrial respiration causes oxidative damage to cellular organelles. It also activates the machinery responsible for mitochondria-dependent apoptosis by releasing apoptotic chemicals from the damaged mitochondria (Haddad and Nakamura 2015). This is most likely the primary way that the neurotoxicant causes cytotoxicity. The presence of FXL noticeably improved the cellular mitochondrial bioenergetics when cells were cotreated with FXL and the pesticide, aligning with previously published data. FXL precursor fucoxanthin (FX) was shown to enhance MCII, III, and V expression when cotreated with palmitate-exposed macrophages, with higher levels of Pgc1a and Tfam revealed that cells cotreated with FX had increased mitochondrial biogenesis but decreased mitochondrial content compared to PA-treated macrophages (Richardson et al. 2005). A plausible rationale for this could be that, even with the diminished mitochondrial pool after FX therapy, greater clearance of malfunctioning mitochondria (mitophagy) may have restored mitochondrial function (Subramaniam and Chesselet 2013). According to experiments conducted on primary hippocampal neurons in rats, FX therapy protected the loss of MMP against ROS challenge and decreased the superoxide buildup in the mitochondria. Additionally, oral FX supplementation raised the levels of DJ- 1 protein in the hippocampus tissues of middle-aged rats, suggesting possible neuroprotective effects against mitochondrial dysfunction linked to ROS (Giordano et al. 2012). Moreover, it was discovered that FX improved MMP, decreased oxidative stress, and activated the AMP-activated protein kinase pathway to improve mitochondrial bioenergetics in palmitate-treated HepG2 cells (Li et al. 2021).

Also, the current data showed that CPF and MPTP significantly increased ROS release and lipid peroxidation with decreased activities of the antioxidant enzymes CAT and SOD and their coding genes expression in addition to inhibition of the expression of the antioxidant genes *Nrf2* and *HO- 1*. FXL cotreatment was shown to decrease pesticide-induced oxidative damage. Recent data showed that FX exhibits antioxidant activities against oxidative stress induced by H_2_O_2_ in human osteoblasts. In cell-free assays, FX extracted from brown algae displayed high levels of DPPH radical scavenging and iron-chelating activities (Ip et al. 2017; Ferdous et al. 2022). FX showed promising antioxidant activities in various in vitro cell lines, including macrophages, liver cells HepG2, epithelial cells of colorectal adenocarcinoma Caco- 2, and human cervical cancer cells HeLa cells. This led to a concentration-based antioxidant effect with a 3.3-fold increase in the antioxidant glutathione level (Yang et al. 2024). Pruccoli et al. (2024) have examined FX and FXL’s overall antioxidant capacity regarding their power to scavenge free radicals. At the same 5 μM concentration, they found that the radical scavenging activity of FXL was noticeably more remarkable than that of FX. It is well known that the two hydroxyl groups in FX's ring structure can transfer electrons or hydrogen atoms, which results in the carotenoid’s antioxidant and free radical scavenging properties (Pangestuti and Kim 2011). Thus, three hydroxyl groups rather than the two groups in fucoxanthin may be responsible for the increased scavenging action shown by FXL (Sachindra et al. 2007).

Our study’s oxidative stress marker changes shed light on oxidative damage in PD and other neurodegenerative illnesses (Compagnoni et al. 2020). Targeting oxidative stress, a key contributor to mitochondrial dysfunction, neuronal damage, and dopaminergic cell death in PD, represents a promising therapeutic approach. This suggests that medicines that reduce oxidative damage, strengthen mitochondria, and promote cellular repair may delay neurodegeneration. Hence, combinations of antioxidants (e.g., N-acetylcysteine, Coenzyme Q10), mitochondrial enhancers (nicotinamide riboside, creatine), and anti-inflammatory drugs may give a more comprehensive neuroprotective impact. To accomplish neuroprotection, research suggests targeting interconnected networks such mitochondrial bioenergetics, oxidative damage, genotoxicity, neuroinflammation, protein misfolding, in addition to the defective autophagy. Thus, comprehensive approach for future PD and neurodegenerative disease management will be more effective.

Our investigation found that FXL successfully prevented apoptosis in the pesticide-treated cells. Pesticide-induced mitochondrial dysfunctions lead to decreased MMP and altered mitochondrial membrane integrity with release of cytochrome c and triggering of caspases apoptosis pathways. Also, genes control the process of planned cell death, known as apoptosis. In response to different apoptotic stimuli, cells upregulate the production of Bax, which inhibits Bcl- 2 and starts the mitochondrial apoptosis pathway. This causes the mitochondria to release cytochrome c into the cytoplasm, which triggers caspase- 9 and caspase- 3 to cause programmed cell death. Nevertheless, apoptosis can be inhibited by overexpressing Bcl-xL or Bcl- 2, stopping cytochrome c from being released. Bax and Bcl- 2, two members of the Bcl- 2 family of proteins, are essential for controlling programmed cell death; Bax stimulates programmed cell death, while Bcl- 2 impedes it. FXL treatment mitigates these effects by maintaining mitochondrial integrity, reducing ROS accumulation, and enhancing antioxidant activity, which prevents apoptosis by downregulating Bax and upregulating Bcl- 2. By stabilizing mitochondria and interrupting apoptotic signaling. Hence, FXL emerges as a promising therapeutic candidate for neurodegenerative conditions. Similar outcomes were reported by Zeng et al. (2018), who showed that FX and FXL could inhibit apoptosis in the liver HepG2 cell line caused by Tributyltin. Furthermore, FX was also found to protect against H2O2-induced apoptosis in the monkey kidney fibroblast cell line (Vero) by reducing the occurrence of apoptotic bodies, indicating its potential to shield cells against atomic fragmentation during oxidative stress (Heo et al. 2008). FXL inhibition of apoptosis may be due to its ability to inhibit mitochondrial disruption and oxidative damage, as both mechanisms can activate apoptosis pathways and pathways related to the gene’s family (Sinha et al. 2013).

Interestingly, the current study findings are in accordance with the suggested bioinformatics finding regarding the importance of mitochondrial, oxidative damage and apoptosis in CPF and MPTP-induced neurotoxicity, and the protective effect of FXL through interacting positively with the suggested pathways. The current molecular docking study identified key molecular targets involved in oxidative stress regulation (Nrf2, HO- 1, and catalase), apoptosis (Bax and Bcl2), mitochondrial function (ND1, ND5, CO1, and cytochrome B), which align with the mechanisms observed in our in vitro experiments. The stronger binding affinity of FXL to these neuroprotective targets, compared to MPTP and CPF, further supports its potential role in mitigating neurotoxicity. Overall, our docking results provide molecular evidence supporting the mechanisms observed in vitro, reinforcing the neuroprotective potential of FXL through its interactions with key targets regulating oxidative stress, mitochondrial function, apoptosis, and inflammation.

Several compounds, such as resveratrol, curcumin, and N-acetylcysteine, ursolic acid, and Withania somnifera herb have been reported to counteract oxidative stress and apoptosis in dopaminergic neurons, primarily by scavenging reactive oxygen species (ROS) (Prakash et al. 2014; Rai et al. 2016; Rai and Singh 2020; Theofanous and Kourti 2022). However, our study demonstrates that FXL provides robust neuroprotection not only by scavenging ROS but also by significantly reducing mitochondrial oxidative stress and apoptosis, key contributors to dopaminergic neurodegeneration in PD. Unlike resveratrol and curcumin, which primarily modulate oxidative stress and inflammation, FXL exhibits a distinctive ability to preserve mitochondrial function by stabilizing mitochondrial membrane potential and inhibiting apoptotic pathways. Additionally, while some polyphenolic compounds suffer from limited bioavailability (Aatif 2023), FXL, as a direct metabolite of fucoxanthin, demonstrates improved bioactivity and stability, further enhancing its therapeutic potential (Pruccoli et al. 2024). Its dual action in mitigating both MPTP- and CPF-induced toxicity highlights its broader protective scope against multiple neurotoxic insults, positioning it as a strong candidate for PD management. These findings underscore the need for further investigation into FXL’s mechanistic pathways and its potential translational applications in neuroprotection.

### Study Limitations

This study explored various cytotoxic mechanisms such as bioenergetics, oxidative damage, and apoptosis to understand the effects of CPF and MPTP on DA cells. However, other potential cellular pathways and mechanisms as inflammation, autophagy, and genotoxicity may not have been thoroughly explored. Another limitation was that the data was based on in vitro models, which may not fully replicate the complexity of in vivo bone environments. Using a human cell line may minimize the effect of interspecies differences but conducting other in vivo experiments to validate the in vitro findings is recommended. Additionally, a single maintained secondary cell line instead of primary cells derived from multiple donors can be considered a limitation. However, this secondary cell line provides a larger population of cells with reproducible results and minimizes the ethical issues associated with using primary cells. Furthermore, the concentration of CPF and MPTP used, and the exposure durations might not cover all possible levels and time frames that could occur in real-world scenarios. Another critical challenge is drug delivery and bioavailability. Ensuring that FXL can effectively cross the blood–brain barrier (BBB) and reach target neurons at therapeutic concentrations will require further pharmacokinetic and pharmacodynamic studies. Optimizing the formulation, such as using nanoparticle-based delivery or prodrug strategies, may enhance FXL’s brain penetration and therapeutic potential. Additionally, long-term safety and dosing strategies need to be carefully evaluated. Hence, it is recommended to evaluate FXL in well-established PD animal models, such as the MPTP-induced mouse model, which closely mimics dopaminergic neurodegeneration observed in human PD. These studies will assess FXL’s effects on behavioral outcomes, dopaminergic neuron survival, mitochondrial function, oxidative stress markers, and neuroinflammatory responses. Additionally, biodistribution and pharmacokinetic studies will be conducted to determine FXL’s ability to cross the blood–brain barrier and its stability in vivo.

## Conclusion

The researched neurotoxic substances CPF and MPTP were found to harm the specialized DA cells. CPF and MPTP also notably reduced ATP levels and activities of mitochondrial complexes to varying degrees while simultaneously increasing cellular lactate production. All examined oxidative stress markers displayed changes consistent with CPF and MPTP’s ability to provoke oxidative stress. FXL was observed to effectively counteract the toxic effects of CPF on the DA neurons, suggesting potential therapeutic use in managing Parkinson’s disease. Cell viability assays confirmed that FXL did not induce significant cytotoxicity at the concentrations used, as cell viability remained comparable to vehicle-treated controls. However, we acknowledge the importance of evaluating long-term effects and higher-dose toxicity in future studies to further establish the safety profile of FXL for potential therapeutic applications. However, we acknowledge the importance of evaluating long-term effects and higher-dose toxicity in future studies to further establish the safety profile of FXL for potential therapeutic applications. These studies will help bridge the gap between experimental models and human exposures, thereby strengthening the translational potential of our research. In addition, direct comparative studies are needed to evaluate FXL’s neuroprotective potential. Its multifaceted approach suggests it may offer comparable or even enhanced neuroprotection through its combined effects on mitochondrial bioenergetics and quality control. Future investigations comparing FXL with other neuroprotective compounds in terms of efficacy, dose–response, and long-term benefits would be valuable in positioning FXL as a potential therapeutic candidate.

## Supplementary Information

Below is the link to the electronic supplementary material.

Supplementary file1 (DOCX 44 kb)

## Data Availability

Data of this study are available from the corresponding author upon reasonable request.
