# Supplementary material for: Fucoxanthinol Mitigates the Cytotoxic Effect of Chlorpyrifos and MPTP on the Dopaminergic Differentiation of SH-SY5Y Human Neuroblastoma Cells
Source: J Mol Neurosci. 2025 Apr 8;75(2):46. doi: 10.1007/s12031-025-02342-7 (PMC11978686; doi:10.1007/s12031-025-02342-7)

**Table S1.** Primers used in the real-time polymerase chain reaction assays of the study

| **Gene** | **Sense (5′-3′)** | **Antisense (5′-3′)** |
| --- | --- | --- |
| ***ND1*** | ACACTAGCAGAGACCAACCGAA | GGGAGAGTGCGTCATATGTTGT |
| ***ND5*** | CTATCTCGCACCTGAAACAAGC | GGTGGAGTAGATTAGGCGTAGG |
| ***Cy.b*** | TATTCGCCTACACAATTCTCCG | GCTTACTGGTTGTCCTCCGATT |
| ***CO1*** | TACGTTGTAGCCCACTTCCACT | GGATAGGCCGAGAAAGTGTTGT |
| ***ATP 6/8*** | CCATCAGCCTACTCATTCAACC | GCGACAGCGATTTCTAGGATAG |
| ***GAPDH*** | GACAGTCAGCCGCATCTTCT | GCGCCCAATACGACCAAATC |
| ND1: NADH dehydrogenase subunit 1, ND5: NADH dehydrogenase subunit 5, Cy.b: cytochrome B, CO1: cytochrome C oxidase subunit 1, ATP 6/8: ATP synthase subunit 6/8, GAPDH: glyceraldehyde-3-phosphate dehydrogenase**.** | | |

Figure S1: Suggested genes interacting with chlorpyrifos (CPF) according to "Comparative Toxicogenomics Database (CTD) using <https://ctdbase.org/>" (last accessed November 9, 2024)


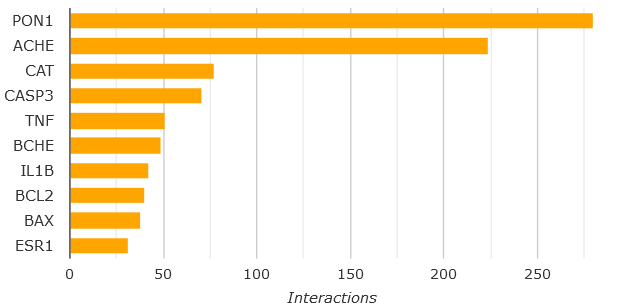


Figure S2: Suggested genes interacting with MPTP according to "Comparative Toxicogenomics Database (CTD) using <https://ctdbase.org/>" (last accessed November 9, 2024)


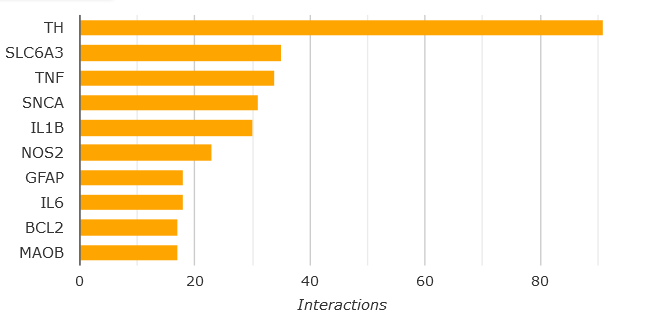


Figure S3: Suggested genes interacting with fucoxanthin (the parent flavonoid for fucoxanthinol) according to "Comparative Toxicogenomics Database (CTD) using <https://ctdbase.org/>" (last accessed November 9, 2024)


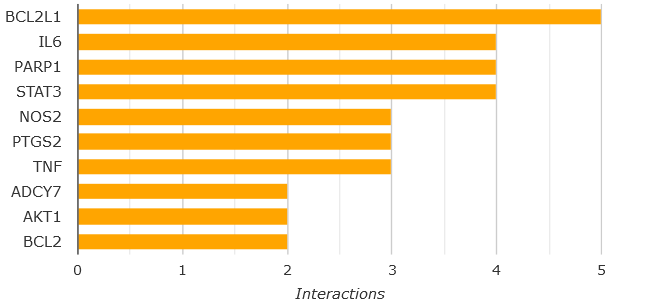

Supplement: Supplementary file 1 — Supplementary file1 (DOCX 44 kb) [file 12031_2025_2342_MOESM1_ESM.docx]
